# Design and Fabrication of Low-Temperature 3D-Printed Bioactive Polyurethane/MnO_2_ Scaffolds for Bone Repair

**DOI:** 10.3390/polym17233101

**Published:** 2025-11-22

**Authors:** Long Li, Along Guo, Yangyi Nie, Zili Xu, Junjie Deng, Yuyang Zhang, Zhenyu Yao, Wei Zhang, Yuxiao Lai, Yuanchi Zhang

**Affiliations:** 1 Centre for Translational Medicine Research & Development, Shenzhen Institutes of Advanced Technology, Chinese Academy of Sciences, Shenzhen 518055, China; lilong@siat.ac.cn (L.L.); al.guo@siat.ac.cn (A.G.); yy.nie@siat.ac.cn (Y.N.); zl.xu2@siat.ac.cn (Z.X.); jj.deng2@siat.ac.cn (J.D.); zyuyang211@163.com (Y.Z.); zy.yao@siat.ac.cn (Z.Y.); yx.lai@siat.ac.cn (Y.L.); 2 University of Chinese Academy of Sciences, Beijing 101408, China; 3 National Innovation Center for Advanced Medical Devices, Shenzhen 518131, China; 4 Key Laboratory of Biomedical Imaging Science and System, Chinese Academy of Sciences, Shenzhen 518055, China; 5 Guangdong Engineering Laboratory of Biomaterials Additive Manufacturing, Shenzhen 518055, China

**Keywords:** low-temperature 3D-printed scaffolds, polyurethane, MnO_2_, responsive release, bone repair

## Abstract

Bone defect repair presents a significant clinical challenge, especially for critical-sized defects, due to the limitation of conventional 3D-printed scaffolds to provide simultaneous mechanical support and bioactivity. Herein, this study developed a bioactive composite scaffold through a low-temperature rapid prototyping (LT-RP) 3D printing technology. The scaffold comprises a polyurethane (PU) matrix enhanced with bioactive manganese dioxide (MnO_2_) nanoparticles, combining structural integrity with versatile bioactivity for bone repair. By incorporating 2, 6-pyridinedimethanol (PDM) into the PU molecular network, a coordination system is formed, enabling homogeneous distribution and structural integration of MnO_2_ nanoparticles. As designed, the bioactive scaffolds are fabricated through LT-RP 3D printing technology with a regular porous architecture for improving cell growth. With 10 wt% MnO_2_, the scaffolds (PPM10) have optimal comprehensive properties, with a modulus of ~14.1 MPa, improved thermal stability, good cytocompatibility, and enhanced osteogenic differentiation. Furthermore, in vitro degradation tests revealed the responsive release of Mn^2+^ from the PPM10 scaffolds in a glutathione-rich microenvironment. This functionality indicates the potential of the scaffolds to modify the tumor microenvironment for ultimate bone regeneration after bone tumor surgery.

## 1. Introduction

Bone defects resulting from trauma, tumors, or surgical excision represent major challenges in orthopedic clinicals [1,2,3]. Although autografts and allografts remain the gold standard for bone repair, limitations including inadequate donors, irregular shape, and inflammatory response actuate researchers to develop new bioactive implants for enhancing bone regeneration [4,5,6]. Synthetic biomaterials including metals, ceramics, and polymers have garnered increased interest to provide mechanical support and facilitate new bone formation. However, traditional metallic implants may suffer from stress-shielding effects and require secondary surgery for removal, while ceramic materials often lack the toughness required for load-bearing applications [7,8]. Polymers offer tunable properties, such as adaptable strength and good biocompatibility. These advantages make them increasingly popular for preparing bone repair implants [9,10,11]. In addition, macroscopic and microscopic structures of implants significantly influence their mechanical and bioactive properties, as well as materials–tissues interactions [12,13]. These factors ultimately determine the efficacy of bone regeneration [14,15]. In recent years, additive manufacturing, commonly known as 3D printing, has revolutionized polymer-based tissue regeneration. This technology enables the fabrication of patient-specific scaffolds with tailored architectures and controlled porosity [16,17]. The porous scaffolds can further facilitate cell infiltration and tissue integration. Among various 3D printing techniques, extrusion-based methods such as fused deposition modeling (FDM) and direct ink writing (DIW) have been widely adopted for constructing biomimetic structures [18,19,20]. However, conventional high-temperature printing processes often limit the incorporation of thermosensitive bioactive molecules, growth factors, or functional nanoparticles, as well as disrupt their physical and chemical properties, which are essential for pro-osteogenic performance of the scaffolds.

To address these issues, low-temperature rapid prototyping (LT-RP) 3D printing technology has emerged as a promising alternative. This approach allows for the processing of biomaterials under mild conditions. It thereby preserves the bioactivity of incorporated nanofillers and enables the use of polymers susceptible to degradation or loss of functionality at high temperatures. Furthermore, this approach constructs scaffolds with precise pores and surface roughness for enhancing cell attachment and proliferation, as well as vascularization and nutrient transport. In previous work, our group has developed a series of LT-RP 3D-printed bioactive scaffolds for bone regeneration [21,22,23]. In particular, polyurethane (PU) has garnered attention as a versatile polymer for biomedical applications because of its exceptional versatility, good mechanical properties, and biocompatibility [24,25,26,27]. Moreover, PU possesses a unique molecular structure composed of hard and soft segments. This distinctive architecture allows personalized physical and biological properties to be tailored for specific clinical requirements [28,29]. Recent research has reported that the PU and PU-based composites can be applied for cardiovascular repair, skin wound repair, and hard tissue repair [30]. When combined with bioactive nanoparticles, PU-based composites can effectively promote osteoconduction and osteogenesis [31]. Manganese dioxide (MnO_2_) has recently been explored as a bioactive nanofiller due to its osteogenic, angiogenic, and antitumoral properties [32,33]. Manganese ions (Mn^2+^) have been shown to promote osteogenic differentiation of mesenchymal stem cells and enhance angiogenesis, both critical processes for successful bone repair [34,35]. Incorporating MnO_2_ nanoparticles into a polymer matrix can effectively improve a scaffold’s functionality by supporting cell adhesion, proliferation, and differentiation. Moreover, MnO_2_ may contribute to mechanical reinforcement and introduce antioxidant or anti-inflammatory effects [36,37]. In addition, due to the acidic environment and high glutathione (GSH) in the tumor microenvironment, MnO_2_ can be reduced and decomposed into Mn^2+^, enabling subsequent highly anti-tumor performance through activating the immune system [38,39,40]. However, a general physical blend of bioactive nanoparticles within a PU matrix might disturb its molecular structures. This can reduce the ion release efficiency and compromise mechanical properties, further hindering ultimate bone regeneration.

**Figure 1:**
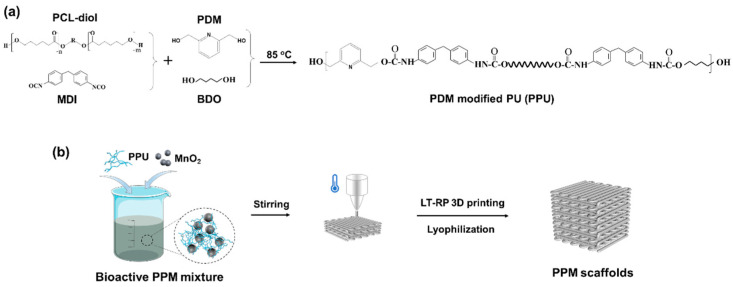
Design and fabrication of the low-temperature 3D-printed bioactive scaffolds. (**a**) Synthesis of the PPU. (**b**) Preparation of the scaffolds.

In this study, we designed and fabricated a PU/MnO_2_ composite scaffold using LT-RP 3D printing technology. The PU matrix was first synthesized using polycaprolactone (PCL)-diol as the soft segment, and the 4,4′-methylenebis (phenyl isocyanate) (MDI), 1, 4-butanediol (BDO) and 2, 6-pyridinedimethanol (PDM) as the hard segment (Figure 1a). There are two -OH groups and the pyridine ring in the PDM molecule. On one hand, the -OH ensured the reaction between the MDI and the PDM. On the other hand, the N atom of the pyridine ring can easily form coordination and hydrogen bonds with metal ions and the H atom of urethane, respectively [41,42]. In this work, due to the modification of the PDM, the Mn ions (Mn^2+^) from MnO_2_ nanoparticles could form a coordination system to integrate the polymer molecular network for achieving comprehensively improved properties. In addition, we uniformly mixed the PDM modified PU (PPU) and MnO_2_ nanoparticles in the 1, 4-dioxane solution, followed by fabricating the bioactive scaffolds using the LT-RP 3D printing method (Figure 1b). As a proof of concept, the scaffold was comprehensively investigated. The assessment included structural characteristics (macro/micro), mechanical and thermal properties, specific degradability and ion release profiles, as well as biological performance in vitro, specifically cytocompatibility and osteogenic differentiation.

## 2. Materials and Methods

### 2.1. Synthesis of the PPU

PPU was synthesized based on our previous studies [1,9]. Briefly, dried PCL-diol (Mn ~4500, Sigma-Aldrich Co., Ltd., Shanghai, China) and MDI (Sigma-Aldrich Co., Ltd., Shanghai, China) were mixed in tetrahydrofuran (THF, Macklin Biochemical Technology Co., Ltd., Shanghai, China) solution and stirred mechanically at 85 °C for 2–3 h. Then, BDO (Sigma-Aldrich Co., Ltd., Shanghai, China) and PDM (J&K Scientific, Beijing, China) were also mixed in THF with a molar ratio of 1:1, followed by being added to the oligomer solution for further reaction. Upon completion of the reaction, the final mixture was quickly poured into a polytetrafluoroethylene mold and placed in an 80 °C oven to cure for 16 h, resulting in the PPU products.

### 2.2. Fabrication of the Low-Temperature 3D-Printed Scaffolds

At first, the PPU and MnO_2_ nanoparticles (60–100 nm, J&K Scientific, Beijing, China) were added into a 1, 4-dioxane (Shanghai Ling Feng Chemical Reagent Co., Ltd., Shanghai, China) solution with various ratios (0 wt%, 2 wt%, 10 wt%, 15 wt%, and 20 wt%, relative to the PPU weight) and stirred uniformly to form the bioink. The detailed composition can be found in Table S1. The scaffolds were then fabricated under a low temperature of ~−30 °C using An22 LT-RP 3D printing machine (CLRF-2000-II, Tsinghua University, Beijing, China). The printing room of the machine was cooled down to −30~−25 °C for 2–3 h in advance. And the print speed was 26.4 mm s^−1^; the extrusion speed was 1.61 mm^3^ s^−1^; the nozzle diameter was 600 µm; the layer height was 0.14 mm; the line distance was 1.10 mm; and the inner line distance was 0.10 mm. Then, the porous scaffolds were processed into a vacuum lyophilizer (Bo Yi Kang FD-1-50, Beijing, China) for removing solvent to obtain final PPU and PPU with various MnO_2_ nanoparticles (PPM2, PPM10, PPM15, and PPM20) scaffolds.

### 2.3. Characterization of the 3D-Printed Scaffolds

The morphologies of the scaffolds were recognized using a scanning electron microscope (SEM, ZEISS SUPRA^®^ 55, Carl Zeiss, Oberkochen, Germany) and energy-dispersive X-ray spectroscopy (EDS, X-Max 20, Oxford, UK). The dry weight and volume of the scaffold sample was first measured and recorded as W_0_ and V. Then the scaffold was immersed in the alcohol solution for 2 h. After that, the scaffold was taken out and weighed to determine its weight (recorded as W_1_). The porosity P for each sample was calculated according to the following equation: P = (W_1_ − W_0_)/ρ_alcohol_/V × 100%. Fourier transform infrared (FTIR) spectrometer (Frontier, Perkin-Elmer, Waltham, MA, USA) with attenuated total reflectance accessories was used to investigate the structure of the scaffolds in the wavenumber range of 500–4000 cm^−1^. The molecular weight of the synthesized PPU samples was characterized by gel permeation chromatography (GPC, Agilent 1260, Waldbronn, Germany). ^1^H NMR measurement of the PPU sample was carried out using an NMR spectrometer (SSNMR, Avance III 400 MH, Zurich, Switzerland). The wide-angle X-ray diffraction (XRD) patterns of the scaffolds were scanned from 2θ = 10° to 80° using an X-ray diffractometer (D8 Advance, Bruker, Karlsruhe, Germany) with a Cu Ka radiation source (1.54 Å). The oxidation state of the MnO_2_ nanoparticles in the scaffold was characterized by an X-ray photoelectron spectroscopy (XPS, Thermo Scientific™ Nexsa™, Brno, Czech Republic). The measurement was performed using an Al Kα X-ray source (λ = 0.83 nm, hν = 1486.6 eV) operated at 72 W. Thermal properties of the samples were measured using a differential scanning calorimetry (DSC) (Mettler Toledo, TGA/DSC1, Zurich, Switzerland) and a thermogravimetric analysis (TGA) machine (Mettler Toledo, DSC1), respectively. The temperature range was −20 to 150 °C with a temperature rate of 10 °C min^−1^ in DSC tests and −30 to 800 °C, with the same temperature rate in TGA tests. The compression measurements were conducted using an electronic universal testing machine (WANCE, Shenzhen, China) at a testing rate of 1 mm min^−1^ at room temperature. The scaffolds were compressed to the maximum deformation of ~80% for obtaining stress–strain curves, followed by calculating the modulus and stress for comparison. The compressed scaffold was then fractured in liquid nitrogen, followed by investigating the cross-sectional morphologies by the SEM (Zeiss Sigma300, Göttingen, Germany).

### 2.4. In Vitro Bioactivity of the 3D-Printed Scaffolds

Cell viability and osteogenic differentiation of the rat bone marrow mesenchymal stem cells (rBMSCs, under an original passage number of 3–5) cultured with extract liquid of the scaffolds were investigated in this work. Cells cultured in medium without extract liquid were used as the control group. Following sterilization with gamma irradiation (15 kGy for 3 h, JPY ION-TECH. Co., Ltd., Shenzhen, China), the scaffolds were first immersed in α-minimum essential medium (α-MEM, Gibco, Grand Island, NY, USA) supplemented with 10% (*v*/*v*) fetal bovine serum (FBS, Gibco, USA) and 1% (*v*/*v*) penicillin/streptomycin (Gibco, USA). The extraction was performed at a fixed mass-to-volume ratio of 0.1 g mL^−1^ for 24 h at 37 °C in a 5% CO_2_ atmosphere. The rBMSCs were cultured using the extract liquid of the scaffolds in 6-well culture plates with a density of 3 × 10^4^ cells well and a density of 4 × 10^4^ cells well, respectively. After 7 days, the Live/Dead staining and alkaline phosphatase (ALP) staining assays were conducted to investigate the cell viability and osteogenic differentiation of the rBMSCs, as with our previous study [9]. Then, the stained cells were observed and recorded by an inverted fluorescence microscope (OlympusCK-2, Tokyo, Japan). The rBMSCs were also co-cultured with the scaffolds for 24 h. After being fixed with 4% paraformaldehyde, the cytoskeletal and nuclei of the rBMSCs were stained using Phalloidin (C2203S, Beyotime, Shanghai, China) and DAPI (C1005, Beyotime), respectively. A laser scanning confocal microscope (LSM 880, Zeiss) was used to record cell morphologies.

### 2.5. Evaluation of Degradation of the PPM Scaffolds

The PPM10 scaffolds were used to investigate the degradation and Mn^2+^ releasing properties in the glutathione (GSH)-containing phosphate-buffered saline (PBS) solution. This is due to the frequent overexpression of GSH in the tumor microenvironment, accompanied by a weakly acidic pH value. Therefore, MnO_2_ can react with the GSH in the tumor microenvironment to responsibly generate Mn^2+^ via the following reaction [43]:

MnO_2_ + 2GSH + 2H^+^ → Mn^2+^ + GSSH + 2H_2_O

This process could modify the tumor microenvironment to promote ultimate bone regeneration. The samples were processed to a ~10 × 10 × 10 mm cube and the initial weights were recorded as *M*_0_. Then the samples with a ratio of 0.1 g mL^−1^ were immersed in PBS solution with different concentrations of GSH (0.5, 1, 2, 6, and 10 mM), and then placed in a thermostatic shaking water bath bed (70 rpm, 37 °C) for a certain period. At each time point, the weights of the samples were measured and recorded as *M_t_*. Therefore, the ratio of residual weight (%) could be calculated by the equation of *M_t_* × *M*_0_^−1^ × 100%. In addition, the extract liquids were collected at each time point. Then, the concentrations of Mn^2+^ in each group were measured by inductively coupled plasma mass spectrometry (ICP, Agilent 710, Shenzhen, China) to further calculate cumulative release of Mn^2+^ from the PPM scaffolds in the PBS solution with various GSH concentrations.

### 2.6. Statistical Analysis

Quantitative data are indicated as the mean ± standard deviation (SD). Two-tailed Student’s *t*-test was used for statistical analyses. There were no adjustments made for multiple comparisons. *p* < 0.05 was assumed to be a statistically significant difference between the compared groups. Quantitative results were respectively analyzed using the software Origin 2022, SPSS 27.0 and Microsoft Excel 2013.

## 3. Results and Discussion

### 3.1. Characterization of the Low-Temperature 3D-Printed Bioactive Scaffolds

**Figure 2:**
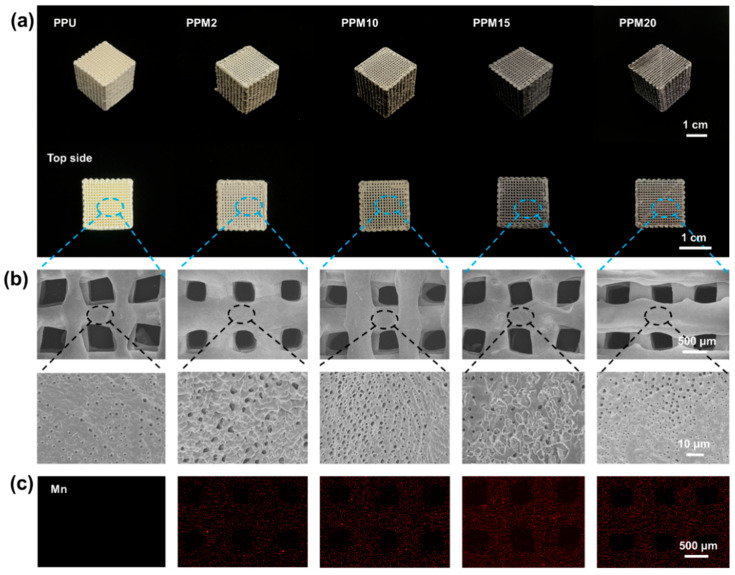
Macroscopic and microscopic structures of the scaffolds. (**a**) Macroscopic images of the scaffolds and their topside views. (**b**) Microscopic morphologies observation with different magnifications of the scaffolds by SEM. (**c**) The element composition distributed in the scaffolds by SEM-EDS. Red: Element Mn.

The structures of 3D-printed bioactive scaffolds comprised PPU and various MnO2 nanoparticles (PPU: 0 wt%, PPM2: 2 wt%, PPM10: 10 wt%, PPM15: 15 wt%, and PPM20: 20 wt%) were first characterized. With increasing content of the MnO_2_ nanoparticles, the color of the scaffolds became darker, while the PPU was almost white (Figure 2a). Topside view of the scaffolds showed regular porous structures at macroscopic scale. Moreover, SEM images indicated the surface morphologies of the pores (diameter: ~400–600 µm) with random roughness that was largely attributed to the generation of microscopic pores throughout the lyophilization of the scaffolds (Figure 2b and Figure S1). The porosities of the 3D-printed scaffolds were also proved to exceed 85% (Table S2). The macroscopic and microscopic pores and roughness have been proven to support cell growth in our study [9]. In addition, the scaffolds were identified by EDS that MnO_2_ nanoparticles were homogeneously distributed inside (Figure 2c).

**Figure 3:**
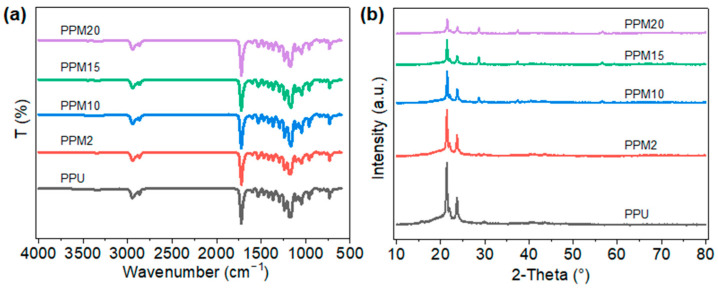
Characterization of the scaffolds. (**a**) FTIR spectra. (**b**) XRD results.

Figure 3a shows the FTIR spectra of the PPU and its composite scaffolds. Specifically, the characteristic bands at 1720 cm^−1^ represented the stretching vibration of the C=O groups in PU matrix. The absorption vibration of the benzene ring structures appeared at 730 cm^−1^. Due to the introduction of the PDM, the characteristic bands at 1605 cm^−1^ and 1535 cm^−1^ represented the absorption of N-H bending vibration and the stretching vibration of N-H in amide II from the PDM molecules, respectively. There was no characteristic bond at around 2270 cm^−1^, proving the NCO groups in the PPU and its composites have been completely reacted. The PPU sample exhibited a number-average molecular weight (M_n_) of approximately 5.16 × 10^4^ g/mol, with a polydispersity index (PDI) of 2.5143 (Figure S2). ^1^H NMR spectrum of the PPU sample confirmed the successful incorporation of both PDM and BDO into the polyurethane backbone (Figure S3). Figure 3b presents the XRD results of the PPU and its composite scaffolds. All patterns exhibit crystalline peaks near 2θ = 21.4° and 2θ = 23.7°. These peaks corresponded, respectively, to the reflection of (110) and (200) planes of PCL within the scaffolds [27]. MnO_2_ crystalline peaks appeared near 2θ = 28.6°, 2θ = 37.50°, and 2θ = 56.65°, with the peak at 2θ = 28.6° being the strongest. After incorporation of the MnO_2_ nanoparticles, the typical peaks appeared at 2θ = 28.6° in the corresponding XRD patterns of the composite scaffolds, while no peak was in that of the pristine PPU scaffolds. Moreover, with the increase in the MnO_2_ in the scaffolds, the intensity of the peaks became larger. Meanwhile, excessive nanoparticles might increase the interplanar spacing of PCL crystallization in the composite, resulting in the weaker peaks at 2θ = 21.4° and 2θ = 23.7° in the XRD patterns of the PPM15 and PPM20 scaffolds compared to those of other scaffolds. XPS spectrum of the PPM10 scaffolds suggested the binding energy difference between the two split peaks of Mn 3s was 4.98 eV, indicating that the dominant Mn valence in the scaffold was Mn^4+^ of the MnO_2_ (Figure S4) [44,45]. These results demonstrated that the PPU has been successfully synthesized and the 3D-printed PPM scaffolds have also been prepared well.

### 3.2. Thermal Properties of the Low-Temperature 3D-Printed Bioactive Scaffolds

**Figure 4:**
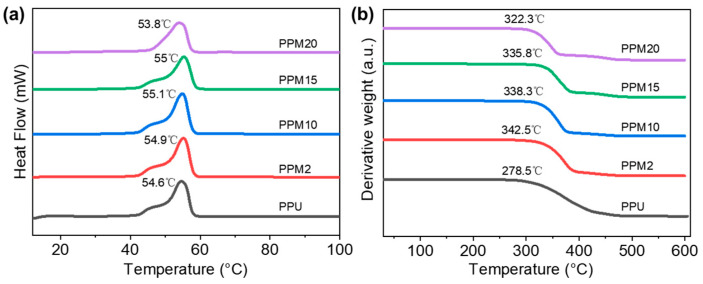
Thermal properties of the scaffolds. (**a**) DSC and (**b**) TGA results.

DSC and TGA were performed to investigate the thermal properties of the scaffolds. DSC results indicated that the melting temperature (T_m_) of the PPU (~54.6 °C), PPM2 (~54.9 °C), and PPM10 (~55.1 °C) scaffolds first increased with the higher content of the MnO_2_ nanoparticles (Figure 4a). This might be attributed to the formation of the coordination systems between the MnO_2_ and the polymer chains in PPU. Nevertheless, the PPM15 and PPM20 scaffolds had, respectively, a decreased T_m_ of 55 °C and 53.8 °C, which might be caused by the excessive introduction of the nanoparticles. TGA curves showed that the decomposition temperature of the PPU scaffold was ~278.5 °C, while those of MnO_2_-incorporated samples increased to above 300 °C, proving the enhanced thermostability due to the integrated structures in the 3D-printed bioactive scaffolds.

### 3.3. Mechanical Properties of the Low-Temperature 3D-Printed Bioactive Scaffolds

**Figure 5:**
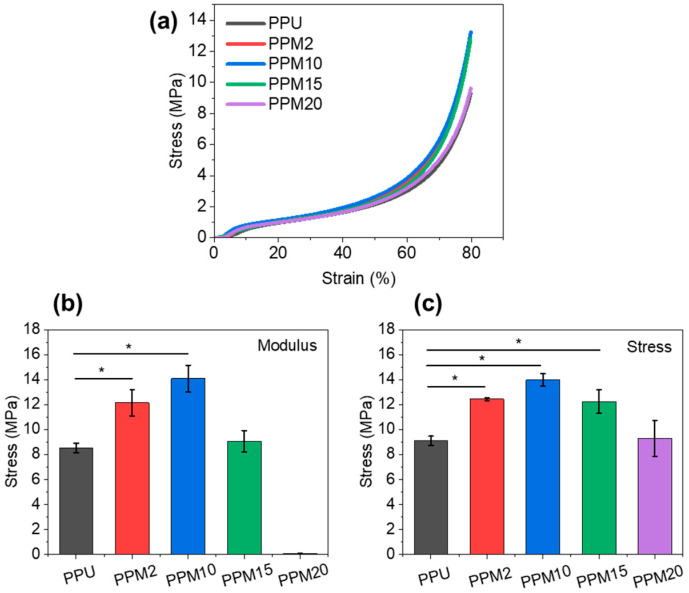
Mechanical properties of the 3D-printed scaffolds. (**a**) Stress–strain curves. (**b**) Modulus and stress at 80% elongation (**c**) derived from (**a**). *, significant difference compared to PPU, *p* < 0.05.

Compression tests on the scaffolds were conducted to evaluate their mechanical performance (Figure 5a). The PPU scaffolds had a modulus of ~8.5 MPa and a stress at 80% of ~9.1 MPa (Figure 5b,c). In comparison, PPM2 and PPM10 scaffolds had significantly improved modulus and stress, especially the PPM10 scaffolds with a modulus of ~14.1 MPa and a stress at 80% elongation of ~13.8 MPa. These should be caused by the reinforcing effect of the nanoparticles and the formation of the coordination systems among the polymer chains and nanoparticles. Although PPM15 scaffolds still had a higher strength than the PPU scaffold, the modulus and stress were lower than those of the PPM2 and PPM10 scaffolds. As for the PPM20 scaffolds, their mechanical properties further decreased due to the destruction of structures and possible stress concentration by excessive MnO_2_ nanoparticles. In addition, the cross-sectional morphologies of the fractured PPM10 scaffold suggested that the structures of scaffolds could be disturbed after undergoing a large deformation while the micropores remain on the surface (Figure S5). These results also confirmed the above structural and thermal analysis of these 3D-printed bioactive scaffolds.

### 3.4. In Vitro Cell Studies

**Figure 6:**
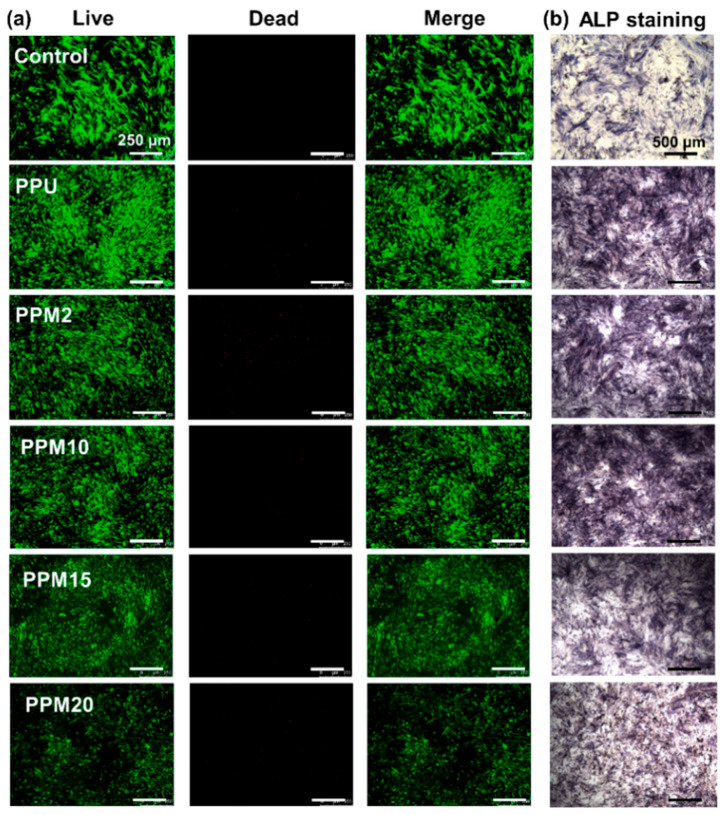
In vitro cell studies of the 3D-printed bioactive scaffolds. (**a**) Fluorescence images of rBMSCs after 7 days incubation in various groups. Green: live cells. Red: dead cells. (**b**) ALP staining images of rBMSCs after 7 days incubation in various groups.

The effects of the scaffolds on cell viability and osteogenic differentiation were evaluated in this work. At first, Figure 6a displayed the fluorescence images of rBMSCs incubated with various samples’ extract liquid for 7 days using the Live/Dead staining assay. Compared with the control group, there was no difference among the PPU, PPM2, PPM10, and PPM15 groups, proving good cell viability in these groups. However, the dead cells could be found to increase in the PPM20 group, indicating high concentrations of Mn^2+^ might have negative effects on cell viability. Cytoskeleton staining images of the rBMSCs co-cultured with the scaffolds exhibited well-spread morphologies, indicating favorable cytocompatibility of these porous scaffolds (Figure S6). In addition, alkaline phosphatase (ALP) is an enzyme produced during osteoblast differentiation, which serves as an early marker for assessing osteogenic differentiation. In Figure 6b, ALP staining images of the BMSCs incubated in all groups indicated the purple colonies, suggesting good ALP secretion in all groups. Moreover, the purple color in stained colony of the PPM10 group was darker than those of other groups, which might be attributed to the appropriate Mn^2+^ released from the PPM10 scaffolds to further enhance osteogenic differentiation of rBMSCs. Therefore, the PPM10 scaffolds were selected for further experiments.

### 3.5. Degradation and Mn^2+^ Releasing Properties

**Figure 7:**
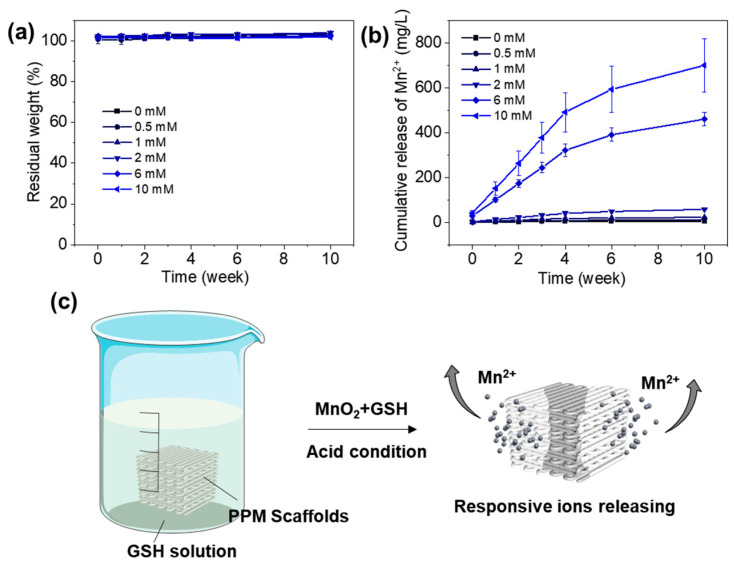
In vitro degradation of the PPM10 scaffolds in the PBS solution with various concentrations of GSH (0, 0.5, 1, 2, 6, and 10 mmol/L). (**a**) Residual weight of the scaffolds during degradation. (**b**) Cumulative release of Mn^2+^ within 10 weeks. (**c**) Schematic illustration of the responsive Mn^+^ releasing of the scaffolds.

Based on the above results, the 3D-printed PPM10 scaffolds had stable thermal properties and significantly enhanced modulus. In addition, the PPM10 scaffolds have also been demonstrated to obtain the pro-osteogenic functions for bone regeneration. Furthermore, we conducted in vitro degradation experiments to investigate the Mn^2+^ releasing behaviors through immersing the PPM10 scaffolds into the PBS solution with various concentrations of GSH for 10 weeks. As shown in Figure 7a, the weight of the scaffolds had no significant changes after being immersed in the solution for 10 weeks, indicating superior stability of the scaffolds for providing long-term support. During the degradation period, the release of Mn^2+^ from the scaffolds was low and slow in the PBS solution without adding GSH or with low concentrations of GSH (0.5, 1, and 2 mM) (Figure 7b). In the PBS solution with higher GSH concentrations (6 and 10 mM), the PPM10 scaffolds released Mn^2+^ rapidly in the first 4 weeks and released sustainably from 4 to 10 weeks. Specifically, the cumulative release amount of the Mn^2+^ in the PBS with 10 mM GSH at 10 weeks was ~130 times more than that in PBS without GSH. This was because the MnO_2_ could react with the GSH under acid condition, then responsively generate Mn^2+^ and change the acid microenvironment (Figure 7c). Given the reported antitumor effects of Mn^2+^ in recent studies, the PPM scaffolds could also be used for the antitumor applications due to the responsive Mn^2+^ releasing functions.

## 4. Conclusions

This work proposed PU/MnO_2_-based bioactive scaffolds through the low-temperature 3D printing method. Refer to the published studies, the PPU modified by introducing PDM molecules enabled the formation of the coordination system among the polymer chains and the MnO_2_ nanoparticles [9,36,41,42]. The 3D-printed PPU and PPM scaffolds had uniformly porous structures with a diameter of 400–600 µm. This structure has been proved to support cell growth for improving bone repair in our previous studies [46,47]. FTIR analysis confirmed the complete consumption of -NCO groups and successful synthesis of the PPU. The results of SEM-EDS, XRD patterns, and XPS results proved the uniform dispersion of MnO_2_ nanoparticles in the scaffolds. In addition, DSC and TGA curves suggested that the introduction of MnO_2_ nanoparticles improved the melting temperature and thermal degradation of the scaffolds, while excessive nanoparticles could reduce their thermostability due to the disruption on the polymer network [48]. Similarly, properly increasing inorganic MnO_2_ achieves higher mechanical properties. Particularly, the PPM10 scaffolds had a modulus of ~14.1 MPa, which can provide adaptive support to the bone defects in agreement with our previous work [9]. In vitro cell studies proved the PPM10 scaffolds had good cell viability and pro-osteogenic effects, which could further enhance bone regeneration. Compared with the pristine PPU scaffolds, the PPM scaffolds with different MnO_2_ nanoparticles had favorable thermal, mechanical, and biological properties. In addition, various publications reported that there was overexpressed GSH with weakly acidic conditions in the tumor microenvironment [38,39,40]. In this work, in vitro degradation of the PPM10 scaffolds in the PBS solution with various concentrations of GSH demonstrated that the scaffolds can responsively release Mn^2+^ in the microenvironment due to the reaction between the MnO_2_ and the GSH. Therefore, the low-temperature 3D-printed PPM10 scaffolds had comprehensively improved properties, suggesting promising potential for practical applications in bone regeneration and antitumor therapy, particularly for repairing post-resection defects following bone tumor surgery.

## Data Availability

Data is contained within the article or Supplementary Materials. Further inquiries can be directed to the corresponding authors.

## Supplementary Materials

The following supporting information can be downloaded at https://www.mdpi.com/article/10.3390/polym17233101/s1, Figure S1: Pore size of the 3D-printed bioactive scaffolds; Figure S2: Molecular weight of the synthesized PPU; Figure S3: ^1^H NMR spectrum of the synthesized PPU; Figure S4: Binding energies of Mn3s in the XPS spectrum of the PPM10 scaffold; Figure S5: The cross-sectional morphologies of the fractured PPM10 scaffold; Figure S6: Cytoskeleton staining images of the rBMSCs co-cultured with the scaffolds. Table S1. The composition of the 3D printed scaffolds. Table S2. The porosity of the 3D printed scaffolds.

## Abbreviations

The following abbreviations are used in this manuscript:

LT-RP	Low-temperature rapid prototyping
PU	Polyurethane
MnO_2_	Manganese dioxide
PDM	2, 6-pyridinedimethanol
PPMx	The scaffolds with x wt% MnO_2_
PCL	Polycaprolactone
MDI	4,4′-methylenebis (phenyl isocyanate)
BDO	1, 4-butanediol
Mn^2+^	Mn ions
PPU	PDM modified PU
SEM	Scanning electron microscope
EDS	Energy-dispersive X-ray spectroscopy
FTIR	Fourier transform infrared
XRD	Wide-angle X-ray diffraction
DSC	Differential scanning calorimetry
TGA	Thermogravimetric analysis
rBMSC	Rat bone marrow mesenchymal stem cells
α-MEM	α-minimum essential medium
FBS	Fetal bovine serum
ALP	Alkaline phosphatase
GSH	Glutathione
PBS	Phosphate-buffered saline
FDM	Fused deposition modeling
DIW	Direct ink writing
