# Supplementary material for: Design and Fabrication of Low-Temperature 3D-Printed Bioactive Polyurethane/MnO2 Scaffolds for Bone Repair
Source: Polymers (Basel). 2025 Nov 22;17(23):3101. doi: 10.3390/polym17233101 (PMC12694104; doi:10.3390/polym17233101)
Supplement: Supplementary file 1 [file polymers-17-03101-s001.zip › polymers-3971720-supplementary.pdf]

polymers

MDPI

Article

Design and Fabrication of Low-Temperature 3D-Printed Bioactive Polyurethane/MnO_2 Scaffolds for Bone Repair

Long Li [1,2,3], Along Guo [1], Yangyi Nie [1], Zili Xu [1], Junjie Deng [1], Yuyang Zhang [1,3], Zhenyu Yao [1,2], Wei Zhang [1,2,*], Yuxiao Lai [1,2,4,5] and Yuanchi Zhang [1,2,*]

[1] Centre for Translational Medicine Research & Development, Shenzhen Institutes of Advanced Technology, Chinese Academy of Sciences, Shenzhen 518055, China; lilong@siat.ac.cn (L.L.); al.guo@siat.ac.cn (A.G.); yy.nie@siat.ac.cn (Y.N.); zl.xu2@siat.ac.cn (Z.X.); jj.deng2@siat.ac.cn (J.D.); zyuyang211@163.com (Y.Z.); zy.yao@siat.ac.cn (Z.Y.); yx.lai@siat.ac.cn (Y.L.)
[2] University of Chinese Academy of Sciences, Beijing 101408, China
[3] National Innovation Center for Advanced Medical Devices, Shenzhen 518131, China
[4] Key Laboratory of Biomedical Imaging Science and System, Chinese Academy of Sciences,
Shenzhen 518055, China
[5] Guangdong Engineering Laboratory of Biomaterials Additive Manufacturing, Shenzhen 518055, China
* Correspondence: zhang.wei@siat.ac.cn (W.Z.); zhangyc@siat.ac.cn (Y.Z.)

polymers

MDPI

Supplementary Information:

Table S1. The composition of the 3D printed scaffolds.

3D Printed Scaffolds	PPU (g)	MnO_2 (g)	MnO_2 (wt%)
PPU	12	0	0 wt%
PPU2	12	0.24	2 wt%
PPU10	12	1.2	10 wt%
PPU15	12	1.8	15 wt%
PPU20	12	2.4	20 wt%

polymers

Table S2. The porosity of the 3D printed scaffolds.

3D Printed Scaffolds	Porosity (%)
PPU	87.55
PPU2	86.37
PPU10	86.26
PPU15	85.66
PPU20	85.34

polymers

MDPI

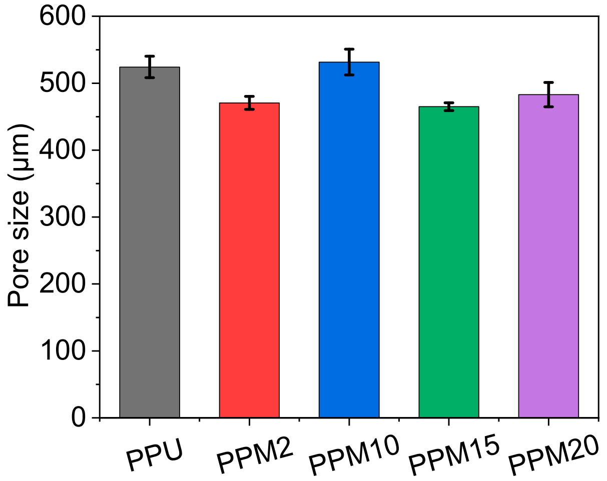

Figure S1. Pore size of the low-temperature 3D printed bioactive scaffolds

polymers

MDPI

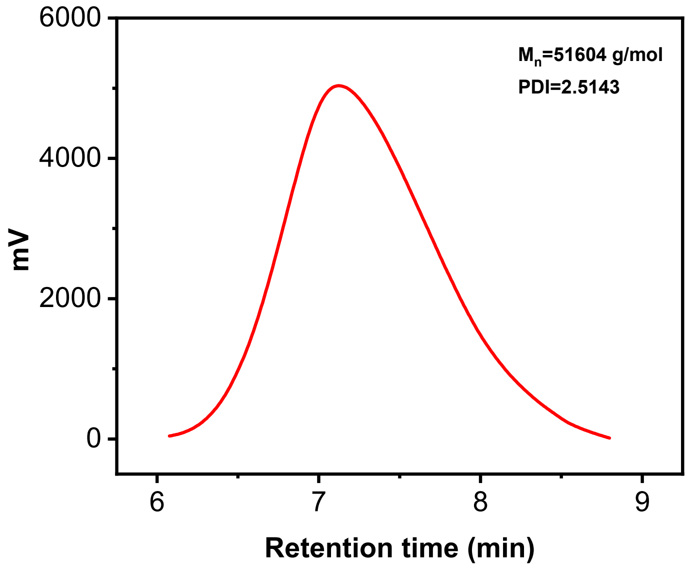

Figure S2. Molecular weight of the synthesized PPU

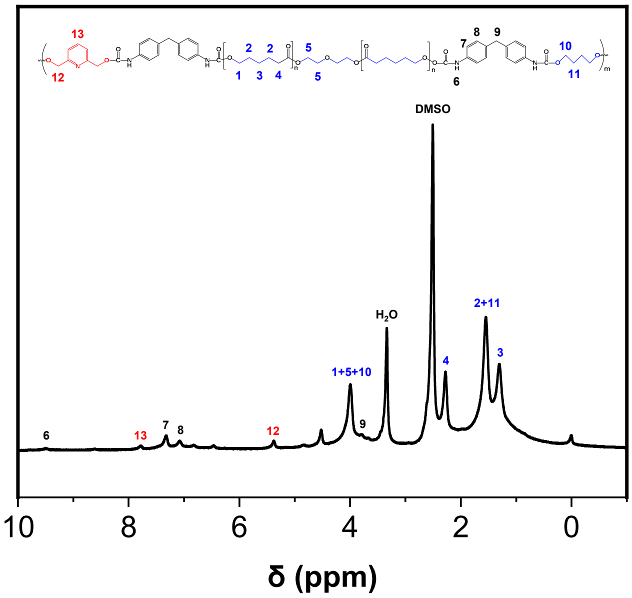

Figure S3. ^{1}H NMR spectrum of the synthesized PPU

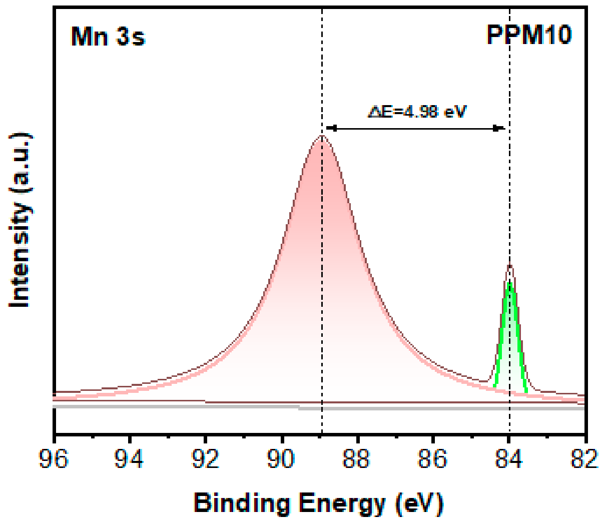

Figure S4. Binding energies of Mn3*s* in the XPS spectrum of the PPM10 scaffold

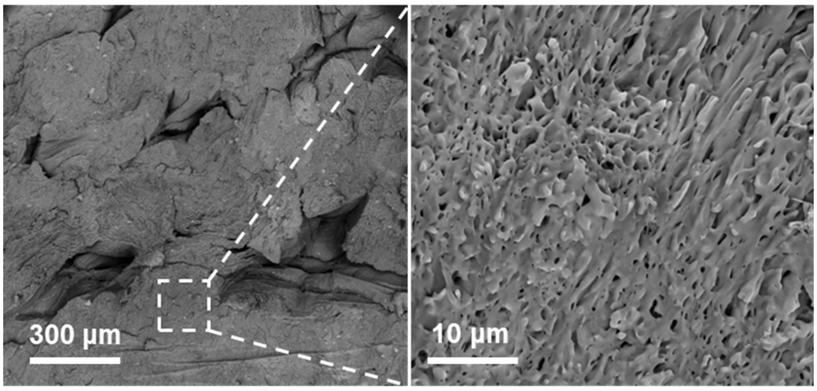

Figure S5. The cross-sectional morphologies of the fractured PPM10 scaffold.

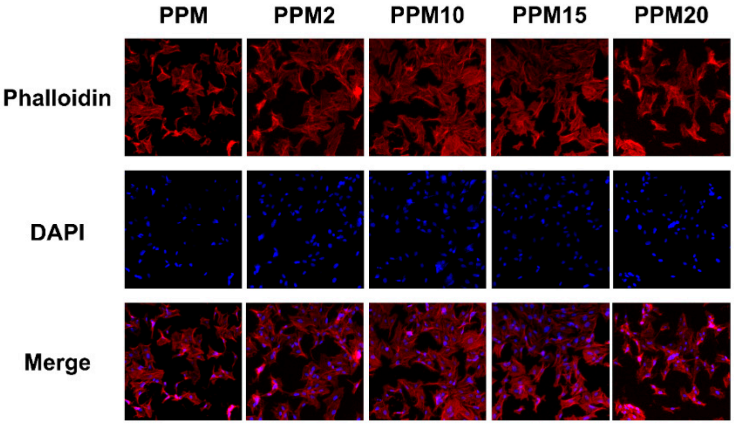

Figure. S6. Cytoskeleton staining images of the rBMSCs co-cultured with the scaffolds.
